# Myths and facts in the treatment of neurodevelopmental disorders – other therapies

**DOI:** 10.1016/j.jped.2025.101452

**Published:** 2025-10-29

**Authors:** Chaves Livio Francisco S., Pessoa Fabio Borges, Marques Pedro Henrique Resende

**Affiliations:** a Departamento de Desenvolvimento e Comportamento da Sociedade Brasileira de Pediatria, Rio de Janeiro, RJ, Brazil; b Fillium Centro Médico, Goiânia, GO, Brazil; c Departamento de Desenvolvimento e Comportamento da Sociedade Goiana de Pediatria, Goiânia, GO, Brasil; d Centro de Referência em Saúde |Mental (CRESM), Goiânia, GO, Brazil

**Keywords:** Neurodevelopmental disorders, Complementary therapies, Myths and facts, Scientific evidence, Non-pharmacological interventions, Alternative therapies

## Abstract

**Objective:**

To critically examine the effectiveness of complementary and alternative therapies (CATs) in the treatment of neurodevelopmental disorders, distinguishing myths from evidence-based practices and supporting informed therapeutic decision-making for healthcare professionals, educators, and families.

**Data sources:**

Evidence was collected from PubMed, Scopus, and Web of Science up to August 2025. Systematic reviews, meta-analyses, and international consensus statements addressing attention-deficit/hyperactivity disorder (ADHD), autism spectrum disorder (ASD), specific learning disorders, communication and language disorders, intellectual disability, and developmental coordination disorder were included.

**Summary of findings:**

Interest in interventions such as neurofeedback, transcranial direct current stimulation, music therapy, equine-assisted therapy, virtual reality, and gamification has grown substantially. However, most of these approaches lack methodological standardization and robust evidence to justify their use as primary treatments. Some demonstrate modest benefits as adjunctive strategies, especially when integrated into structured programs and supervised by multidisciplinary teams. Conversely, therapies including acupuncture, ozone therapy, and hyperbaric oxygen therapy present insufficient scientific support and should not be considered substitutes for validated methods.

**Conclusion:**

Complementary and alternative therapies remain a topic of significant debate in the management of neurodevelopmental disorders. While certain approaches may offer limited adjunctive benefits, their clinical use should be carefully evaluated within evidence-based frameworks. The development of rigorous evaluations, standardized protocols, and the safe integration of innovative technologies is essential to optimize therapeutic outcomes without compromising access to scientifically validated interventions.

## Introduction

Neurodevelopmental disorders comprise a heterogeneous group of early-onset conditions, typically manifesting before school age, characterized by deficits affecting cognitive, behavioral, motor, linguistic, and socioemotional functioning.^1^^,^^2^ The most prevalent conditions include attention-deficit/hyperactivity disorder (ADHD), autism spectrum disorder (ASD), specific learning disorders (SLD), communication and language disorders (CLD), intellectual disability (ID), and developmental coordination disorder (DCD).^1^^,^^2^

The etiology is multifactorial, resulting from the interaction between genetic predisposition, neurobiological alterations, and environmental factors, which together modulate the course and clinical expression of these disorders.^1^ Comorbidities are frequent and increase diagnostic and therapeutic complexity, requiring integrated and individualized approaches for optimal developmental outcomes.^1^^,^^2^

Although pharmacological and psychosocial interventions have established efficacy, interest in non-pharmacological therapies has been increasing, driven by expectations for “more natural” methods, limitations in response to conventional treatments, and the widespread circulation of information—often unvalidated—through digital media.^1^^,^^3^^,^^4^

CATs encompass structured behavioral interventions, digital technologies, motor training programs, non-invasive brain stimulation, and expressive therapies such as music therapy.^3^^,^^5^, ^6^, ^7^ Some have solid supporting evidence, while others lack standardization and replication, necessitating critical evaluation of efficacy and safety.^3^, ^4^, ^5^^,^^8^

The indiscriminate use of unproven methods may delay effective therapies, compromising critical windows of neuroplasticity and functional prognosis. The incorporation of new approaches should be guided by standardized protocols, multidisciplinary supervision, and outcome monitoring.^3^^,^^5^^,^^8^

This article critically reviews recent evidence on CATs for ADHD, ASD, specific learning disorders, communication and language disorders, intellectual disability, and DCD, based on 40 high-quality references (randomized controlled trials, systematic reviews, meta-analyses, and consensus statements), prioritizing those with the highest scientific impact and including essential classic studies.^1^, ^2^, ^3^^,^^5^

### Attention-deficit/hyperactivity disorder

ADHD is a neurobiological disorder characterized by persistent symptoms of inattention, hyperactivity, and impulsivity, negatively impacting academic performance, interpersonal relationships, and emotional regulation.^1^, ^2^, ^3^, ^4^, ^5^, ^6^, ^7^, ^8^, ^9^ Neuroanatomical and neurofunctional alterations in frontostriatal circuits, as well as dopaminergic and noradrenergic dysfunctions, form the pathophysiological basis of the disorder.^1^, ^2^, ^3^, ^4^, ^5^, ^6^, ^7^, ^8^, ^9^ Non-pharmacological interventions play a relevant adjunctive role and, in specific cases, may serve as alternatives.^3^^,^^5^^,^^9^

Digital cognitive training (D-Cog) targeting working memory, selective attention, and inhibition promotes modest gains in neuropsychological measures, with variable transfer to clinical symptoms.^5^^,^^3^ D-Cog, as exemplified by EndeavorRx®, represents a technological innovation approved by regulatory agencies such as the FDA, aimed at improving specific executive functions.^10^ Although randomized controlled trials indicate discrete gains in sustained attention, the evidence does not yet support its standalone use due to inconsistent results and high cost, recommending its integration into multimodal programs.^10^

Neurofeedback, despite growing interest, is hampered by heterogeneous methodologies and a lack of standardization.^11^, ^12^, ^13^, ^14^, ^15^ Double-blind trials and meta-analyses suggest its effect may be equivalent to placebo when applied in isolation, reinforcing the need for its use as part of integrated multimodal programs.^11^^,^^13^, ^14^, ^15^

Mindfulness-based programs, such as MYmind, and online parental interventions demonstrate improvement in emotional regulation and reduction of family stress, achieving efficacy similar to group cognitive behavioral therapy.^16^^,^^17^ Aerobic physical activity is also associated with gains in inhibitory control and reductions in internalizing symptoms, although its recommendation as a standalone ADHD therapy remains insufficiently supported.^16^^,^^17^

Non-invasive brain stimulation, including transcranial direct current stimulation (tDCS) and transcranial magnetic stimulation (TMS), shows potential for modulating cortical excitability and promoting specific benefits in executive functions such as inhibition and processing speed.^7^^,^^18^^,^^19^ However, protocol variability and lack of long-term safety data limit its recommendation for routine clinical use.^7^^,^^18^^,^^19^

In summary, neurofeedback, mindfulness, and digital solutions have stronger empirical support as adjuncts, integrated into individualized, multimodal treatment plans.^5^^,^^9^, ^10^, ^11^^,^^16^, ^17^, ^18^

### Specific learning disorders

Encompassing dyslexia, dyscalculia, and dysgraphia, these disorders are characterized by persistent, specific difficulties in acquiring and automating academic skills, unrelated to intellectual disability or isolated adverse environmental conditions.^20^ The neurobiological basis involves dysfunctions in the neural network responsible for phonological processing, attention, and visuospatial integration.^20^, ^21^, ^22^

The use of colored lenses and Irlen filters has been proposed to modulate visual perception and facilitate reading; however, robust evidence does not support their efficacy in improving reading fluency or comprehension.^23^ tDCS applied to brain areas associated with language has shown promising preliminary results in phonological modulation, although larger and more controlled studies are required for confirmation.^20^, ^21^, ^22^

Therapeutic digital games focused on phonological awareness, working memory, and auditory discrimination may enhance the effects of speech-language and psychopedagogical interventions, reinforcing the need for individualized, supervised approaches.^20^, ^21^, ^22^

Effective management of these disorders requires rigorous diagnosis, intensive and continuous interventions, and multidisciplinary follow-up to prevent associated academic delays and psychosocial impairments [20, 21, 22, 23].

### Autism spectrum disorder

ASD is defined by persistent deficits in social communication and restricted, repetitive behaviors, often accompanied by sensory alterations and cognitive rigidity.^24^^,^^25^ The etiology is complex, involving genetic and environmental factors resulting in dysfunctions across multiple brain networks, particularly in regions associated with sensory integration and socioemotional regulation.^24^^,^^25^ This complexity, coupled with sometimes slow or inconsistent therapeutic responses, has fueled the proliferation of alternative therapies, often marketed with promises of cure or complete rehabilitation.^24^^,^^25^

Among complementary therapies, music therapy and equine-assisted therapy have the strongest scientific support, promoting improvements in social engagement, communication, and motor organization—provided they are integrated into established behavioral training programs such as Applied Behavior Analysis (ABA).^6^^,^^25^^,^^26^

In contrast, practices such as acupuncture, ozone therapy, hyperbaric oxygen therapy, and osteopathy lack sufficient scientific evidence for clinical recommendation and may pose risks or delay established interventions.^6^^,^^25^^,^^26^

Non-invasive brain stimulation, such as tDCS and TMS, shows discrete effects in improving functional communication and emotional regulation; however, the absence of standardized protocols prevents routine use.^27^^,^^28^ Emerging technologies such as virtual reality offer potential for social skills training in adolescents with preserved intellectual functioning, though further studies are needed for broad validation.^7^^,^^27^^,^^28^

Despite CATs, ABA remains the gold standard in international guidelines for ASD intervention and should be prioritized in therapeutic plans.^25^

### Communication and language disorders

CLDs range from expressive language delay to specific language impairment and social communication disorder. They are characterized by persistent deficits in language comprehension and/or expression, negatively affecting learning, socialization, and socioemotional development.^1^^,^^20^ Etiology is multifactorial, including genetic factors, structural and functional neurological alterations, and environmental influences.^19^

Evidence-based speech-language therapy—structured, intensive, and tailored to the patient’s profile—is the treatment of choice.^1^^,^^20^ Complementary approaches, such as digital games targeting phonological and semantic skills, may enhance gains when supervised by specialists.^23^ tDCS applied to cortical areas related to language has shown preliminary positive results, but lacks standardized protocols for routine use.^7^

The use of unproven therapies, such as unvalidated auditory stimulation or alternative methods without a scientific basis, may delay effective interventions and compromise functional prognosis.^14^

### Intellectual disability

Characterized by significant limitations in intellectual functioning and adaptive behavior, ID begins during the developmental period and results from multiple causes, including genetic factors, perinatal insults, and environmental influences.^1^ There are no treatments capable of reversing ID, except in rare situations with treatable etiologies.^1^^,^^29^

The most effective interventions include early stimulation programs focusing on cognitive, social, and adaptive skills.^1^^,^^30^, ^31^, ^32^ ABA has robust evidence for improving adaptive skills and reducing challenging behaviors in individuals with ID, especially when started early and applied intensively.^1^^,^^33^

Assistive technologies, curricular adaptations, and accessibility resources promote school and social inclusion [1]. Virtual reality and gamification-based interventions have shown limited benefits for visuomotor coordination and motivation but still require validation in large-scale RCTs.^33^, ^34^, ^35^, ^36^, ^37^, ^38^

Myths about “curing” ID persist and are often exploited by unproven approaches that divert resources and delay effective therapies.^14^

### Developmental coordination disorder

DCD involves significant difficulties in acquiring and executing coordinated motor skills, affecting activities of daily living and school performance.^1^^,^^29^ Etiology includes dysfunctions in corticospinal and subcortical circuits responsible for motor planning.^1^^,^^29^

Task-oriented training programs, such as Neuromotor Task Training (NTT) and Task-Oriented Motor Training (TOMT), have the strongest evidence of efficacy, especially when integrated with structured physiotherapy and occupational therapy.^4^^,^^29^^,^^30^

Active video games (exergames) may serve as motivational tools for motor training but should not replace proven methods, given methodological heterogeneity and lack of standardization.^29^

Functional electrical stimulation and virtual reality also show specific benefits in coordination and reaction time, but still lack uniform protocols for widespread adoption.^1^^,^^39^^,^^40^

## Discussion

Critical analysis of CATs in neurodevelopmental disorders reveals substantial methodological heterogeneity¹. Some interventions, such as neurofeedback, mindfulness, music therapy, and task-oriented motor training, have evidence supporting their use as adjuncts in specific contexts.^1^^,^^33^^,^^34^^,^^39^

In ADHD, approaches such as digital cognitive training and neurofeedback may provide additional gains when integrated with conventional treatments but do not replace pharmacotherapy and evidence-based psychosocial interventions.^32^, ^33^, ^34^, ^35^, ^36^, ^37^, ^38^^,^^40^

In ASD, music therapy and equine-assisted therapy demonstrate benefits in social engagement and communication and are recommended as complements to programs such as ABA. Unproven approaches like acupuncture, ozone therapy, and hyperbaric oxygen therapy should not be recommended.^7^^,^^24^, ^25^, ^26^, ^27^, ^28^

In SLDs and CLDs, digital technologies and tDCS have potential as auxiliary resources, but their effectiveness depends on integration into validated methods and professional supervision.^1^^,^^7^^,^^20^, ^21^, ^22^, ^23^

In ID and DCD, early structured interventions and task-oriented motor training remain the cornerstones, with emerging technologies considered only as complements until stronger evidence becomes available.^4^^,^^25^^,^^27^^,^^29^^,^^30^^,^^39^

## Conclusion

The use of CATs in managing neurodevelopmental disorders should follow rigorous scientific criteria, prioritizing safety, efficacy, and integration with validated treatments. While some demonstrate relevant benefits as adjuncts, none should replace methods recognized by international guidelines.^1^^,^^40^

Inappropriate application of these therapies may delay effective interventions and compromise prognosis. Therapeutic decisions should be shared among the multidisciplinary team, family, and patient, considering clinical context, functional impact, and available resources.^1^^,^^2^^,^^5^^,^^40^

Public policies and clinical guidelines should direct the careful incorporation of CATs, ensuring equitable access to evidence-based treatments while safely and responsibly exploring the potential of new technologies.^1^^,^^2^^,^^5^^,^^40^

## Data availability statement

The data that support the findings of this study are available from the corresponding author.

## Conflicts of interest

The authors declare no conflicts of interest.
